# Screening and treatment of brain metastasis from papillary thyroid carcinoma: a case series

**DOI:** 10.1186/s13044-023-00146-8

**Published:** 2023-01-11

**Authors:** Le Ngoc Ha, Le Quoc Khanh, Ngo Thi Minh Hanh, Hyo Jung Seo, Mai Hong Son

**Affiliations:** 1 Department of Nuclear Medicine, Hospital 108, Hanoi, Vietnam; 2 Department of Pathology, Hospital 108, Hanoi, Vietnam; 3 BNCT Center, Dawonmedax, Incheon, Republic of Korea

**Keywords:** Papillary thyroid carcinoma, Brain metastasis, I- Whole Body Scan, ^18^F-FDG PET/CT, MRI

## Abstract

**Background:**

The brain metastasis from differentiated thyroid carcinoma (DTC) is a rare condition and its prognosis is poor. The standard protocol for screening and treatment of patients with brain metastases from papillary thyroid cancer (PTC) remains controversial. This report aims to share the experience of a single center in the management of brain metastases from DTC.

**Material and methods:**

Five patients with brain metastases were identified from 5000 patients with DTC attending the department of nuclear medicine, Hospital 108 between 2016 to 2022. The statistical software Statistical Package for Social Sciences (SPSS) 20.0 (SPSS Inc., Chicago, IL, USA) was used to analyze the data.

**Results:**

Five patients with brain metastases from DTC were revealed by MRI, ^18^F-FDG PET/CT with contrast enhancement, and ^131^I-SPECT/CT. The median time of overall survival (OS) was 15 months, ranging from 10 to 65 months. Two out of the five patients underwent surgery, and futher 2 patients were treated with stereotactic surgery (SRS). All patients are still alive.

**Conclusions:**

Brain metastases from DTC are rare. MRI is the preferred imaging mobility to screen brain lesions in DTC. The primary treatment modalities are surgery and SRS.

**Supplementary Information:**

The online version contains supplementary material available at 10.1186/s13044-023-00146-8.

## Introduction

Thyroid carcinoma is one of the most common endocrine cancers worldwide. The prevalence of differentiated thyroid cancer (DTC) accounts for 90% of all thyroid cancer types with a favorable prognosis [1]. However, brain metastases from DTC are rare but sometimes become the cause of mortality [2, 3]. The management of patients with brain metastases from DTC is challenging. The number of retrospective studies with more than 20 patients was limited [4–6]. Characterization of clinicopathological features and the possible impact of radio-iodine, tyrosine kinase inhibitors, surgery, stereotactic radiosurgery, external beam radiotherapy, and whole-brain radiation therapy in patients with brain metastases from DTC have been reported in previous studies [7, 8]. However, there is no standard guideline for managing brain metastases from DTC. We have identified five patients with brain metastases among 5000 patients with DTC attending our center between 2016–2022. This case series aims to review our experience in a single center of managing brain metastases from DTC.

### Case 1

**Fig. 1:**
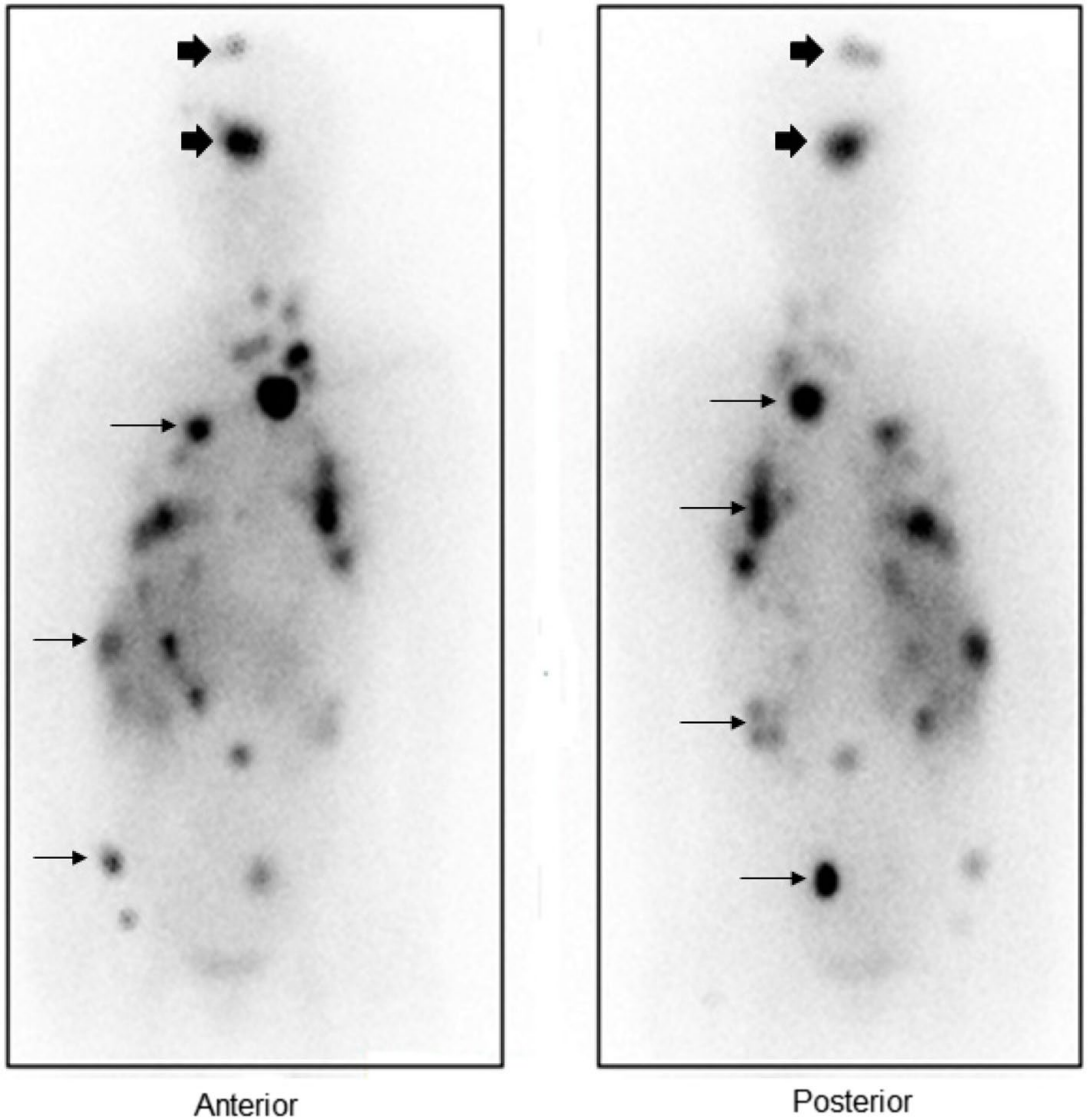
^131^I- post-treatment whole-body scan Focal uptake metastatic lesions localized on the head (arrow head), and metastases in multiple organs (arrows)

**Fig. 2:**
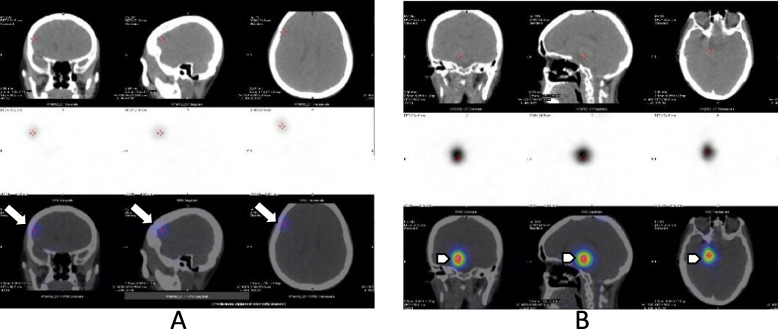
2 mCi.^131^I-SPECT/CT head and neck (**a**): Mild metastatic focal uptake localized in the right frontal lobe (arrow) (**b**): High metastatic focal uptake localized in the right pedicular (arrow)

**Fig. 3:**
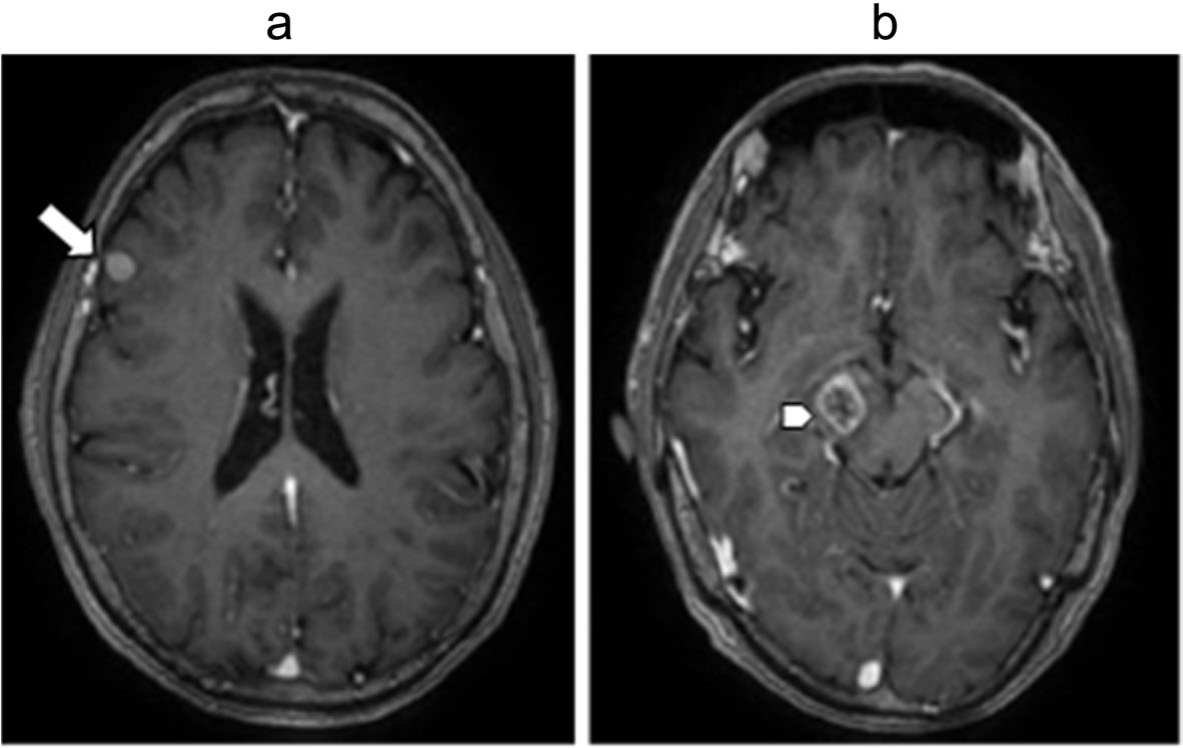
Brain metastases lesions in MRI T1-weighted after contrast enhancement (**a**): The right frontal cortex lesion (arrow). (**b**): The right cerebral peduncule lesion (arrowhead)

A 66 year-old-woman was diagnosed with papillary thyroid carcinoma (PTC), regional cervical and right supraclavicular metastases (pT1N1M1). Diagnostic ^131^I- whole body scan showed multiple focal increased ^131^I-uptake in the head, neck, chest, abdomen, and pelvis, consistent with multi-organ metastases (Fig. 1). At the same time, the patient developed headaches and weakness on the right side, which suggested focal brain lesions. The head and neck ^131^I-SPECT/CT showed uptakes in the focal lesions seen in the ^131^I—whole body scan (Fig. 2). A hypodense lesion measuring 10 mm in diameter in the right frontal lobe and another hypodense lesion measuring 23 × 17 mm in the pons and right cerebral peduncle suggesting brain metastases were identified. She was treated empirically with high dose ^131^I (150 mCi) combined with Dexamethasone (16 mg/day). After the ^131^I treatment, her MRI brain (T1-weighted with contrast enhancement) showed a lesion in the right frontal cortex of 8 mm with focal enhancement and another in the right cerebral peduncle measuring 14 × 17 mm with central necrosis, peritumoral edema and heterogenous enhancement (Fig. 3). The patient underwent stereotactic surgery (SRS) with 24 Gy/1fraction (fx) for the right frontal and 22.5 Gy/3fx for pedicular lesions. After three months, the brain MRI scan showed a partial response tumors with improvement in symptoms. Radioiodine therapy was continued to treat multiple metastatic lesions and follow-up.

### Case 2

**Fig. 4:**
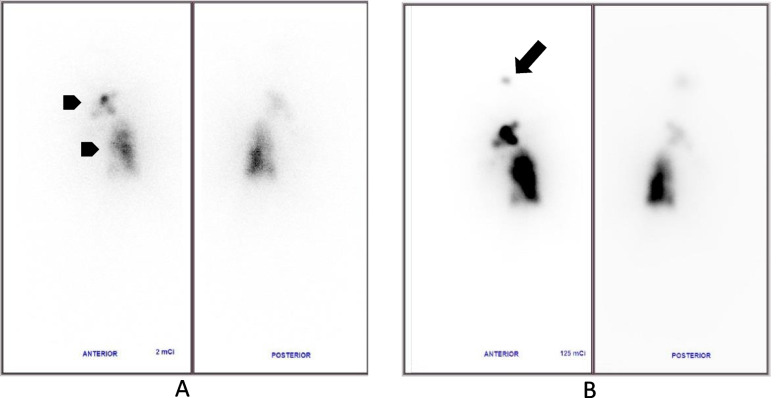
Diagnostic^131^I–whole body scan (**a**) and post-treatment whole body scan (**b**) : Diagnostic ^131^I -whole body scan showing lesions at the thyroid bed and left lung (arrowhead) but no brain metastatic lesions. (**b**): Post-treatment ^131^I -whole body scan showing additional focal uptake on the head (arrow).

**Fig. 5:**
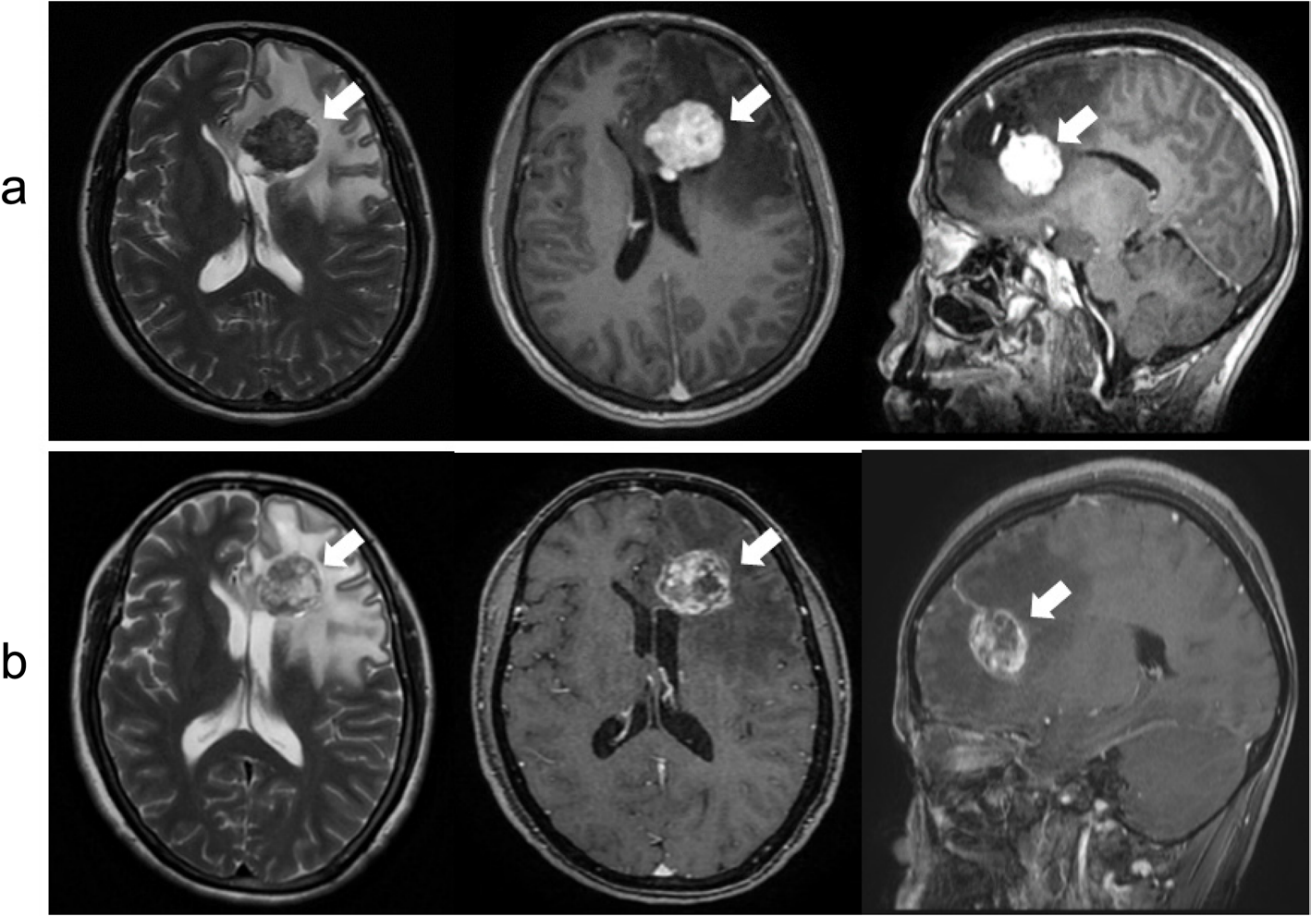
Pre and post radiosurgery Cerebral metastatic lesion (**a**): Pre-radiosurgery MRI showing the left frontal lesion with marked contrast enhancement (upper row). **b**: Three months post-treatment MRI showing no significant change in tumor dimensions but a reduction in contrast-enhanced portion and an increase in the necrotic part (lower row)

A 40-year-old woman presented with dyspnea and a palpable cervical mass. Neck ultrasound showed multiple calcified large thyroid nodules and bilateral neck lymphadenopathy. Chest X-ray also showed multiple opacity ground glass opacities on both lungs. The patient underwent total thyroidectomy and lymph node dissection. Postoperative histopathology confirmed PTC (pT4N1b). Diagnostic ^131^I—whole body scan showed multifocal uptake on the thyroid bed, chest, abdomen and bone, in keeping with multiple metastases (Fig. 4a). An empirical treatment with high dose ^131^I (150 mCi) was given, and a post-therapy ^131^I—whole body scan showed an additional focal uptake on the head (Fig. 4b). Brain MRI revealed a solitary lesion in the left frontal lobe measuring 33 × 31 mm, remarkedly enhanced with peritumoral edema and mass effect causing cingulate herniation, consistent with a brain metastasis. Palliative radiotherapy was given for recurrent lesions on the left thyroid bed and cervical lymph node metastasis. SRS with a dose of 1800 cGy/1fx was used to treat the metastatic brain lesion. The partial response was seen on an MRI performed 3 months after the treatment (Fig. 5). The high empirical dose of radioiodine therapy was continued to treat thyroid beds and lung metastases. The progression-free survival of this patient has been 58 months and the patient is still under follow–up.

### Case 3

**Fig. 6:**
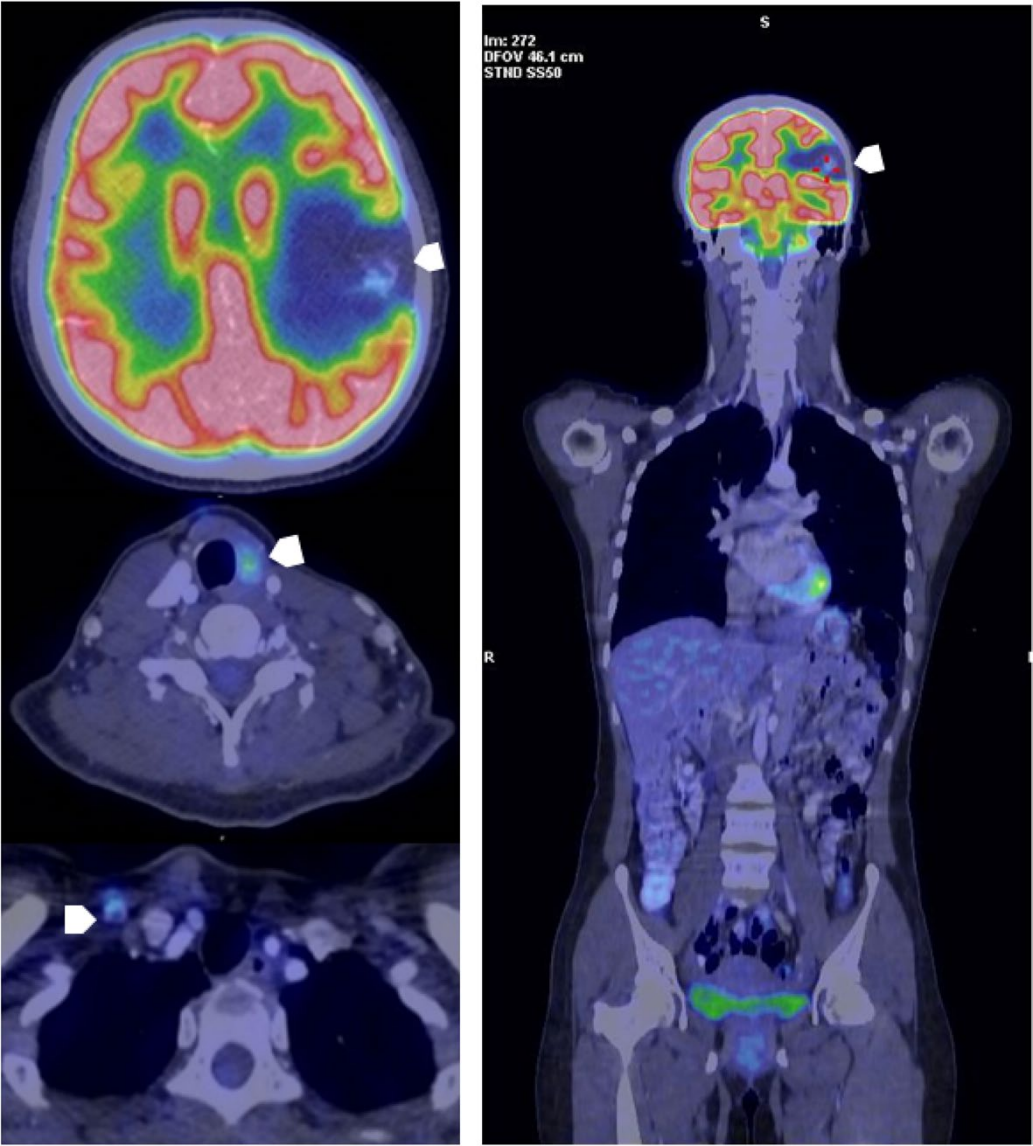
^18^F- FDG PET/CT images A decreased.^18^F-FDG avid metastatic lesion in the left temporal lobe and the thyroid bed recurrent lesions and the right supraclavicular lymph nodes metastases (arrow head)

**Fig. 7:**
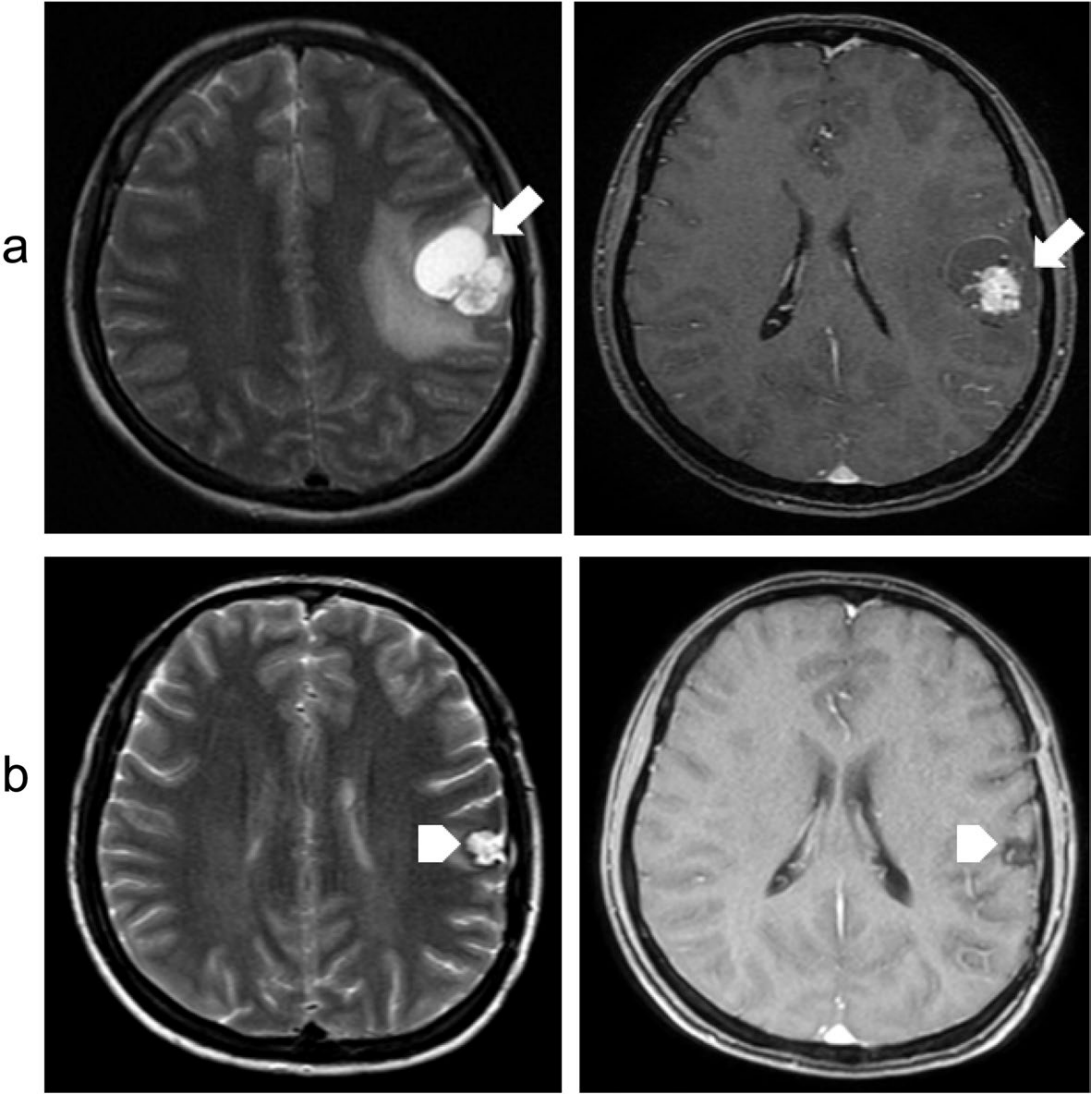
Pre- and post-surgery MRI shows lesions in the left temporal region (**a**): Pre-surgery MRI showed that a cystic portion showed high intensity on T2-weighted and a solid portion showed contrast enhancement on T1 -weighted in the left temporal, and extensive cerebral edema around the tumor (arrow-upper row) (**b**): Three months post-surgery MRI (lower row) shows a lesion with high signal intensity on T2 weighted, low signal intensity without post-contrast enhancement on T1 weighted (arrowheads)

**Fig. 8:**
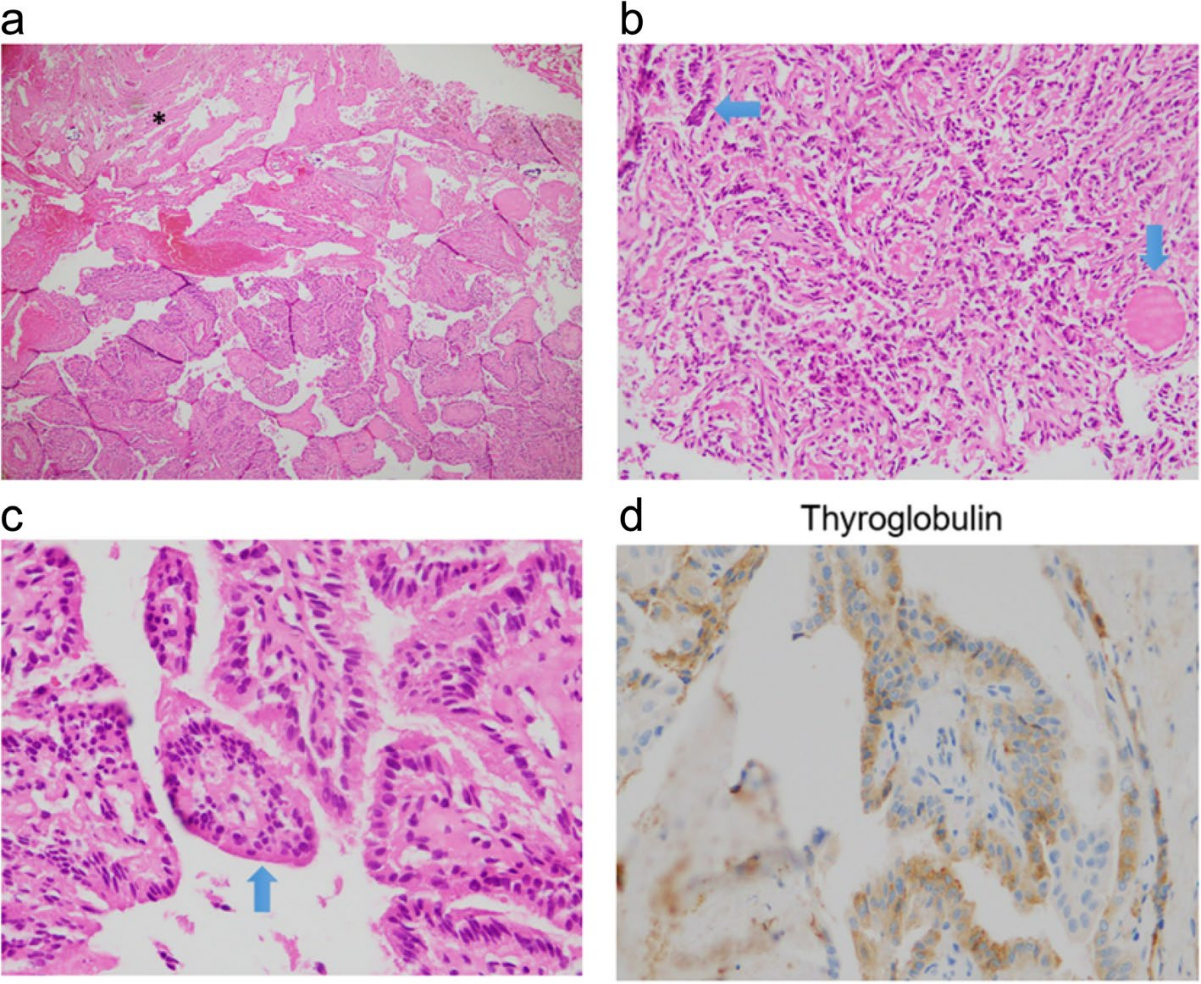
Histopathological and immunohistochemical images of brain metastases Histological images of metastatic papillary thyroid carcinoma (PTC). **a** PTC invades brain parenchyma (1/3 upper left *) (HE stain, × 40), **b** Image suggestive of papillary structureon papillary structure (long arrow), a follicle lined cuboidal cells with dark nucleic regularly contains colloid (short arrow) (HE × 100). **c** The papillary structures are covered by tumor cells without obvious papillary nuclear features (HE stain, × 400). **d** Tumor cells are positive Thyroglobulin (Immunohistochemistry stain, × 400). The histological image showed the majority of papillary structures in the peripheral area of tumor includes only vague papillary structures with edematous, collagenous and fibrous stroma like papillary cores, intercalating secretory follicular structures that contain colloid,. No tumor necrosis was seen and mitosis was rare. Immunohistochemistrical stain shows that tumor cells are positive for Thyroglobulin, TTF1 maker. Ki67 positivity was very low. Nuclear features of papillary thyroid carcinoma were not clear but there was no evidence of poorly differenciated or anaplastic thyroid carcinoma. The histopathological features are consistent with a high-grade papillary thyroid carcinoma

A 33-year-old woman developed PTC with regional lymph node confirmed by ultrasound guided fine-needle aspiration (FNA) and lung metastases on chest CT (cT4aN1bM1). After total thyroidectomy and lymph node dissection, she subsequently underwent radioiodine therapy three times with a total activity of 450 mCi ^131^I since 2018. After the third treatment, post-therapy ^131^I- whole body scan showed no uptake. However, the serum thyroglobulin level was 500 ng/ml. The patient underwent ^18^F-FDG PET/CT for screening for nonradioiodine avid metastases. That showed multiple focal lesions with increased FDG uptake on the thyroid bed, bilateral lymph nodes, and a hypodense mass without FDG avidity, measuring 50 × 46 mm in total diameter on the left temporal lobe (Fig. 6). A brain MRI showed a 38 × 33 mm lesion with cystic and solid component in the left frontal lobe, showing a strong contrast enhancement on T2-weight and hypo signal on T1-weight, consistent with a solitary metastatic focus (Fig. 7a). The large intracranial mass, the thyroid bed lesion, and the bilateral lymph nodes were treated with surgery. The postoperative pathological and immunohistochemical report confirmed that brain metastasis from PTC (Fig. 8). There was no residual brain lesion on MRI after surgery; hence the patient did not undergo adjuvant radiosurgery (Fig. 7b). TSH-suppressive levothyroxine therapy was maintained. The patient has been followed-up for 28 months after the detection of the brain metastases.

### Case 4

A 63-year-old female underwent total thyroidectomy and lymph node dissection, and subsequently followed by remnant ablation and adjuvant ^131^I- therapy with the diagnosis of PTC (cT3N1M0). Post-therapy ^131^I- whole body scan no uptake. Twelve months after the radioiodine treatment, the patient presented to the emergency department with seizures. The head MRI showed a 23 × 25 mm lesion (enhancing on T2-weighted image and co-signal with cortex on T1-weighted image) in the right frontal lobe without peri-tumor edema, consistent with brain metastasis. She underwent a resection of her cranial tumor. Histopathology confirmed brain metastasis from PTC. The patient is under followed-up, and there has been no evidence of progression.

### Case 5

**Fig. 9:**
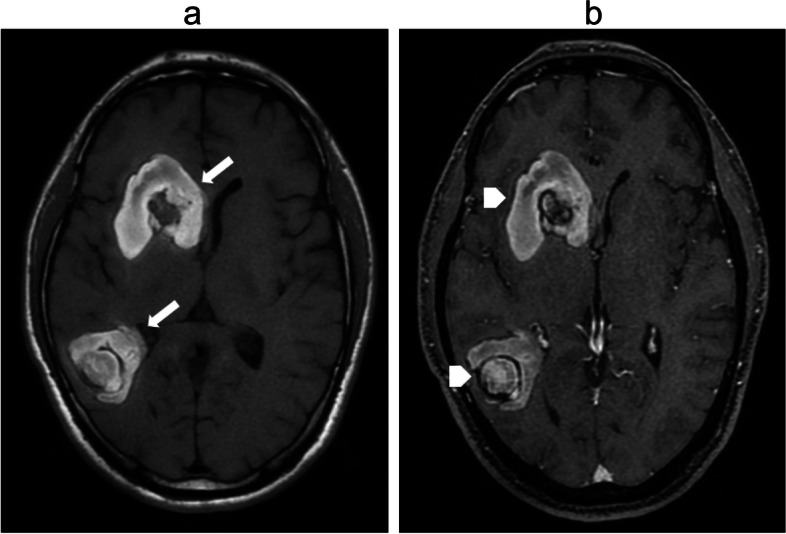
Contrast-enhanced MRI revealed metastatic brain lesions in symptomatic patients (**a**): Lesions at the frontal, right temporal, and lateral ventricles with predominantly high signal intensity on T1-weighted image (arrows). **b** Contrast MRI enhancement strongly infiltrated the centers of the lesions (arrow heads)

A 42-year-old man was diagnosed with PTC and underwent total thyroidectomy, dissection of metastatic neck lymph nodes, and adjuvant radioiodine therapy in 2015. After the third radioiodine treatment, ^131^I- whole body scan was negative with the thyroglobulin level at 300 ng/ml. The patient undertwent ^18^F- FDG PET/CT which showed a sub-centimeter recurrent lesion in the thyroid bed and cervical metastatic lymph nodes at level VI with high FDG avidity. The patient was under follow-up with TSH-suppressive levothyroxine therapy. In March 2021, he presented to the emergency department with persistent severe headache. A non-contrast cranial CT showed hyper-dense lesion at the right caudal nucleus, measuring 13 × 19 mm. In addition, there was a 37 × 38 cm lesion in the right ventricular occipital edge, associated with surrounding cerebral edema consistent with hemorrhage within a metastasis. Brain MRI showed 39 × 43 mm, and 30 × 34 mm solid lesions enhancing brightly on T1-weighted MRI in the right frontal and the right temporal lobe adjacent to the lateral ventricle, respectively. Contrast enhancement strongly infiltrated the centers of the lesions (Fig. 9). Dexamethasone, 4 mg twice a day, was initiated, along with pain control. The patient refused surgery and tyrosine kinase therapy, and he was followed-up. After two months, ^18^F-FDG PET/CT revealed new lesions in the brain, cervical lymph nodes, right scapula and right rectus abdominal muscle with high FDG uptake.

## Materials and methods

Five patients with highly suspicous brain metastases from DTC were identified from 5000 patients with DTC who attended the department of nuclear medicine, Hospital 108 between 2016 to 2022. The criteria for inclusion in this report were: 1) brain metastases from DTC confirmed by histopathology and immunohistochemistry, or 2) highly suspicious brain metastases from DTC on diagnostic imaging, including ^131^I- SPECT/CT, ^18^F-FDG PET/CT, diagnostic CT, or MRI, and 3) patients could be followed up. The medical records were reviewed to collect data on the age, gender, pathology, imaging features of brain metastases, time to detecting brain metastases from the first radioiodine treatment, treatment modalities, and overall survival (OS) for each patient was measured from the date of idenitification of brain metastases to the date of death or the last follow-up visit. This study was approved by the thyroid tumor board at Hospital 108. The patients’ consent forms were waived in view of the retrospective nature of the study.

The statistical software Statistical Package for Social Sciences (SPSS) 20.0 (SPSS Inc., Chicago, IL, USA) was used to analyze the data. The variables not following normal distribution were depicted by median, range, minimum and maximum.

## Results

The prevalence of brain metastases in patients with DTC in our center was 5/5000 (0.1%). There were 1/5 (20%) male and 4/5 (80%) females with a median age of 42 years (range 33–66 years). The median time from the diagnosis of DTC to developing brain metastases was 19.6 months (range 1–63 months) (Table 1). The median time of OS was 15 months (range 10–65 months). Three of the 5 patients with brian metastasis presented with nonspecific symptoms and the other 2 patients were asymptomatic. All patients had histopathology of DTC treated with total thyroidectomy and radioiodine. Three patients presented with brain metastases synchronized with other multiple organ metastases. ^131^I- whole body radioiodine scan showed suspected brain metastases in 2/5 patients. ^18^F-FDG PET/CT detected brain metastases incidentally in another 2/5 (40%) patients. All brain lesions were assessed by contrast-enhanced MRI. Patients with iodine uptake were treated with radioiodine, steroids, SRS (case 1 and 2), and surgery with an image-guided navigation system (case 3 and 4). Two of 5 patients underwent tumor resection surgery and only one patient was followed up without specific intervention. All patients are still alive at the time of completion of the study.

**Table 1:** General clinical characteristics and outcomes of patients with brain metastases

PatientNo	Age at diagnosis of DTC(year)/sex	Histology	Other distant metastases	TNM classification	Time from the initial diagnosis to brain metastasis (months)	Overall survival (months)	Death due to progression
1	66/female	PTC	Lung, bone	T1N1M1	1	39	alive
2	40/female	PTC	Lung	T4bN1M1	1	67	alive
3	33/female	PTC	Lung	T4aN1M1	21	17	alive
4	63/female	PTC	Lung	TxNxM1	12	11	alive
5	42/male	PTC	Soft tissue	T1N1M1	63	11	Progression (alive)

## Discussion

This case series report illustrates the experience in the management of diagnosis and treatment for brain metastases from DTC in our center. Multi-modalities are indicated in the diagnosis and treatment of brain metastases from DTC.

The prevalence of brain metastases from DTC in our center was rare (0.1%), in keeping with published prevalence rates between 0.15 – 1.3% [9–11]. The symptoms of brain metastases are nonspecific, such as headache, focal neurologic dysfunction, cognitive dysfunction, seizures, and stroke [12]. Hence, it is challenging to make a diagnosis based on clinical symptoms. In our report, symptomatic patients were sent for MRI to diagnose brain metastases. However, some brain lesions were incidentally detected by ^18^F-FDG PET/CT and ^131^I-SPECT/CT in asymptomatic patients. MRI is the preferred imaging modality to assess brain metastasis with higher sensitivity and specificity as compared to other modalities [13, 14]. Systemic brain imaging such as MRI, CT, and ^18^F-PET/CT was recommended for screening metastases in radioiodine refractory DTC before tyrosine kinase inhibitor therapy in a multi-center report [15]. ^18^F-FDG PET/CT is a useful tool for screening metastases. In our center, a dedicated head and neck protocol using contrast enhancement was applied to improve sensitivity and specificity in detecting brain and other metastases in patients with high thyroglobulin levels and negative radioiodine scans. However, brain metastases might be found in patients with DTC together with other distant metastases by ^131^I -whole body scan. And ^131^I-SPECT/CT is recommended to confirm the location of brain metastases. Nuclear physicians and oncologists must be aware of the clinico-histopathological heterogeneity of PTC in order to make the early diagnosis of unexpected brain metastasis [16].

The decision-making for treatment of brain metastases in DTC depends on several clinical factors such as systemic tumor burden, histopathology type, radioiodine avidity, and location of the tumor. Four patients in our study benefited from surgery and SRS. Neurosurgical resection seems to be the primary treatment for symptomatic, isolated focal, or less than three tumors with good performance status. Few retrospective studies have confirmed that OS is significantly longer in the resectable brain metastases group in comparison with the unresectable group [17, 18]. Radiotherapy, including whole-brain radiotherapy, SRS, or focussed external beam radiotherapy could be the second option after surgery [19]. Among radiotherapy modalities, SRS is a powerful local treatment modality that can be affective in small, multiple, and deep metastases [20]. SRS has been associated with a higher local control rate and longer OS than those who did not receive this treatment method [21].

Systemic control using tyrosine kinase inhibitors Sorafenib or Lenvatinib combined with radioiodine may improve the progression–free survival and quality of life for patients with radioiodine-refractory DTC [22, 23]. In our study, only one patient was eligible for Sorafenib but he refused the treatment. In differentiated PTC, the role of radioiodine in improving the outcome of brain metastases remains unclear. However, a patient with radioiodine uptake may have a better prognosis than one without radioiodine uptake. Sheu et al. reported that radioiodine combined with other treatment modalities may improve the quality of life in a small number of patients (24). However, the adverse effects of radioiodine therapy were hemorrhage and cerebral edema. In our study, radioiodine combined with TSH suppression and steroids was used to reduce brain tissue edema.

Brain metastases from DTC are associated with poor prognosis. The OS in our study was longer than that in the previous studies [2, 17]. The different results might be related to histopathology. The primary tumor in our study was DTC. Meanwhile, other studies included tumors with more aggressive histopathological types such as anaplastic and medullary thyroid carcinoma. In addition, two patients in our study showed radioiodine uptake in the brain lesions and other distant metastases, and tumors with radioiodine uptake tend to show a better prognosis than tumors with non-radioiodine uptake.

Our study has several limitations. The follow-up period was short and the number of cases was small. Cases of brain metastases were selected from a limited period between 2016 to 2022. The evidence for PTC in the primary tumor was based on histopathological images in 4 out of 5 patients (cases 1–4). We lacked histopathological image for case 5, and the evidence of PTC was based on the histopathological report. Furthermore, we did not use of tyrosine kinase inhibitors for any of our patients with brain metastases from DTC.

## Conclusion

Brain metastases from DTC are rare, and carry a poor prognosis. The screening of brain metastases can be done with MRI, ^18^F-FDG PET/CT with contrast enhancement, and ^131^I- SPECT/CT. The primary treatment modality is surgery for patients with symptomatic, single, or less than three metastatic tumors with good performance status. SRS is indicated for multiple small and deeply located metastatic tumors. Radioiodine may have an additional role in the prediction of prognosis and as a combination therapy with other modalities.

## Supplementary Information

**Additional file 1: Supplementary figure 1.** Histopathological images of primary papillary thyroid carcinoma of case 1. The tumor patterns are composed of complex, branching papillae with fibrovascular cores and follicular architecture (HE stain, low magnification a, b, c, d). Nuclear features were enlargement, elongation, overlapping with irregular contour, and nuclear grooves (HE stain, high magnification e, f). **Supplementary Figure 2.** Histopathological images of the primary papillary thyroid carcinoma (PTC) of case 2 showed multimodule of PTC on the background of thyroid goiter (HE stain x 40, a, b), intercalating fibrous areas, and calcifying patterns (HE stain x 40, c), nuclear features of PTC (HE stain x 200, d). **Supplementary figure 3.** The histological image of brain metastases from PTC (HE stain 40, a, invading to brain tissue, asterisk). The majority of papillary structures covered the tumor peripheral area (blue arrow) and in the center there are fibrous stroma and the secreted follicular structures that contain goiter (white arrow) (HE stain x 40, b). No tumor necrosis was seen and mostly rarity of mitosis. Immunohistochemistrical stain presents that tumor cells are positive with Thyroglobulin (immunohistochemistry stain x 400, d). Nuclear features of papillary thyroid carcinoma were not clear but poorly thyroid carcinoma and anaplastic thyroid carcinoma were no evidence. The images correlate with high-grade papillary thyroid carcinoma. **Supplementary figure 4.** Histopathological image of brain metastasis. Papillary thyroid carcinoma (PTC) including papillary structures invaded the brain parenchyma (HE stain x 100, a, asterisk). The papillary structures were covered by tumors presented features of the PTC nucleus, psammoma is in the center of the papillary score (HE stain x 400, b, black arrow).

## Data Availability

The data that support the findings of this study are available from the corresponding author upon reasonable request.

## Abbreviations

CT: Computed tomography
DTC: Differentiated thyroid cancer
FNA: Fine-needle aspiration
Fx: Fraction
^18^F-FDG: ^18^F-fluoro-2-deoxyglucose
MRI: Magnetic resonance imaging
PTC: Papillary thyroid carcinoma
OS: Overall survival
PET/CT: Positron emission tomography/computed tomography
SRS: Static radiosurgery
SPECT: Single photon emission tomography
