# Supplementary material for: Screening and treatment of brain metastasis from papillary thyroid carcinoma: a case series
Source: Thyroid Res. 2023 Jan 11;16:1. doi: 10.1186/s13044-023-00146-8 (PMC9832656; doi:10.1186/s13044-023-00146-8)
Supplement: Supplementary file 1 — Additional file 1: Supplementary figure 1. Histopathological images of primary papillary thyroid carcinoma of case 1. The tumor patterns are composed of complex, branching papillae with fibrovascular cores and follicular architecture (HE stain, low magnification a, b, c, d). Nuclear features were enlargement, elongation, overlapping with irregular contour, and nuclear grooves (HE stain, high magnification e, f). Supplementary Figure 2. Histopathological images of the primary papillary thyroid carcinoma (PTC) of case 2 showed multimodule of PTC on the background of thyroid goiter (HE stain x 40, a, b), intercalating fibrous areas, and calcifying patterns (HE stain x 40, c), nuclear features of PTC (HE stain x 200, d). Supplementary figure 3. The histological image of brain metastases from PTC (HE stain 40, a, invading to brain tissue, asterisk). The majority of papillary structures covered the tumor peripheral area (blue arrow) and in the center there are fibrous stroma and the secreted follicular structures that contain goiter (white arrow) (HE stain x 40, b). No tumor necrosis was seen and mostly rarity of mitosis. Immunohistochemistrical stain presents that tumor cells are positive with Thyroglobulin (immunohistochemistry stain x 400, d). Nuclear features of papillary thyroid carcinoma were not clear but poorly thyroid carcinoma and anaplastic thyroid carcinoma were no evidence. The images correlate with high-grade papillary thyroid carcinoma. Supplementary figure 4. Histopathological image of brain metastasis. Papillary thyroid carcinoma (PTC) including papillary structures invaded the brain parenchyma (HE stain x 100, a, asterisk). The papillary structures were covered by tumors presented features of the PTC nucleus, psammoma is in the center of the papillary score (HE stain x 400, b, black arrow). [file 13044_2023_146_MOESM1_ESM.pptx]

## Slide 1
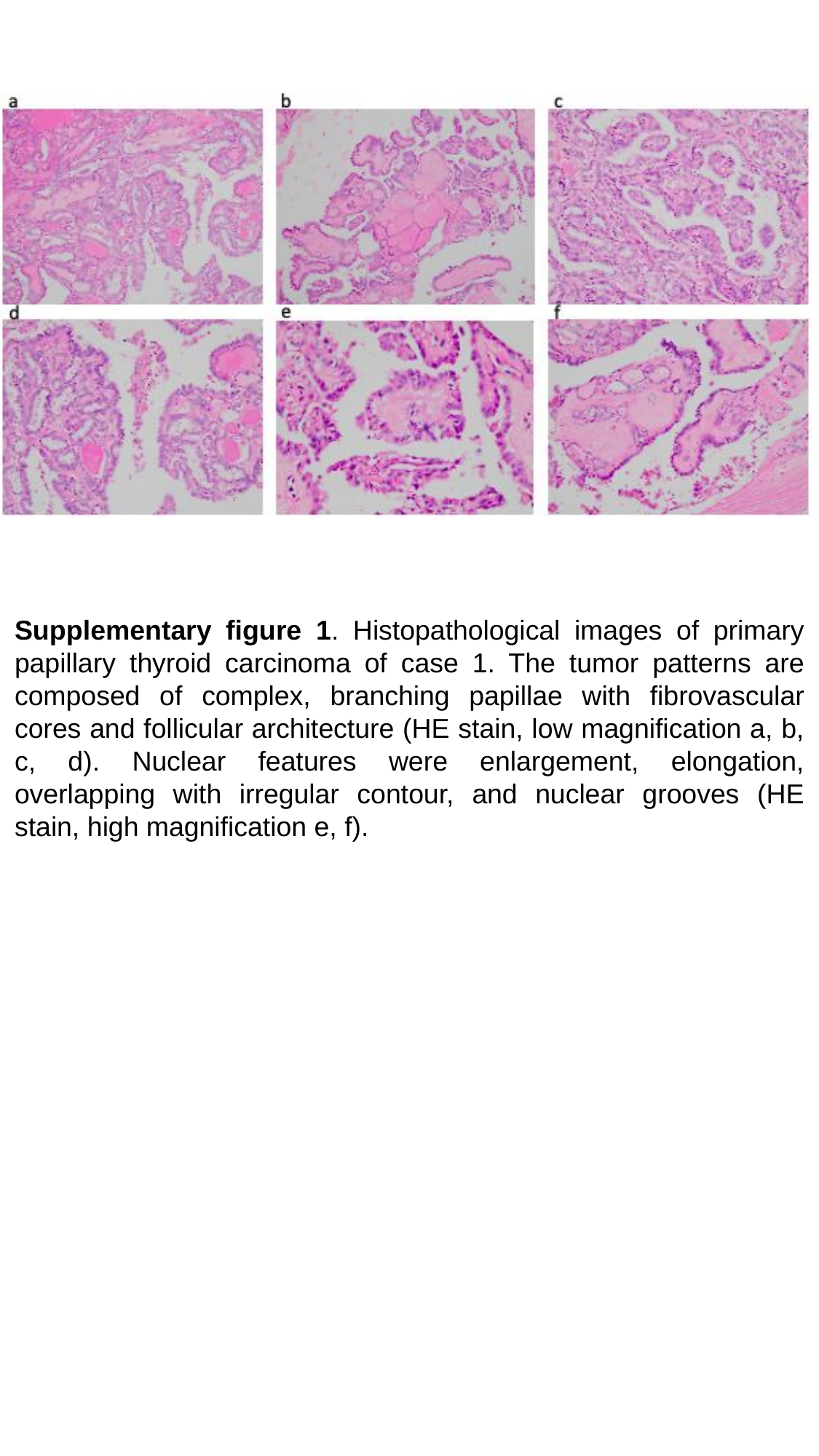

Supplementary figure 1. Histopathological images of primary papillary thyroid carcinoma of case 1. The tumor patterns are composed of complex, branching papillae with fibrovascular cores and follicular architecture (HE stain, low magnification a, b, c, d). Nuclear features were enlargement, elongation, overlapping with irregular contour, and nuclear grooves (HE stain, high magnification e, f).

## Slide 2
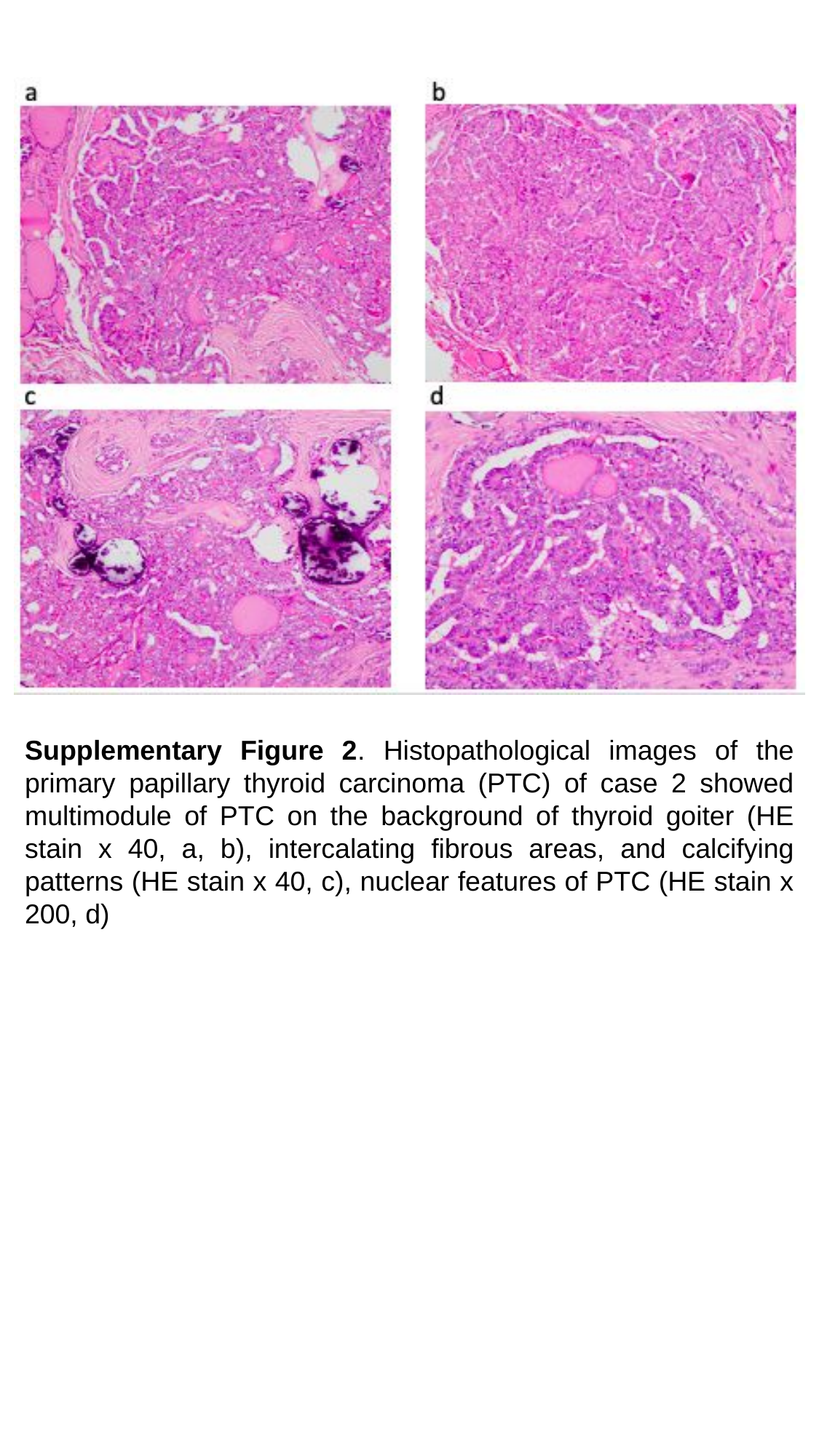

#
Supplementary Figure 2. Histopathological images of the primary papillary thyroid carcinoma (PTC) of case 2 showed multimodule of PTC on the background of thyroid goiter (HE stain x 40, a, b), intercalating fibrous areas, and calcifying patterns (HE stain x 40, c), nuclear features of PTC (HE stain x 200, d)

## Slide 3
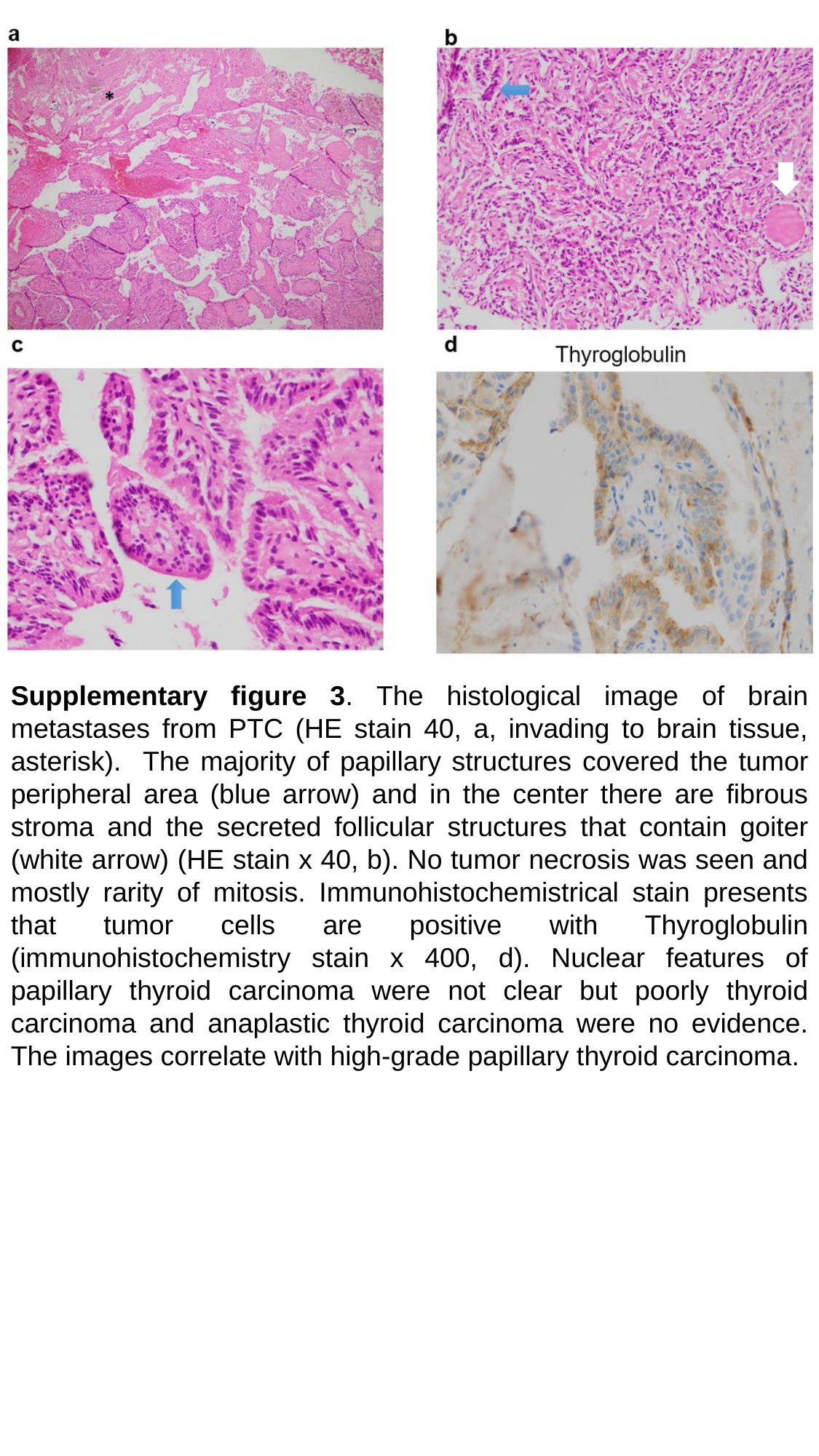

Supplementary figure 3. The histological image of brain metastases from PTC (HE stain 40, a, invading to brain tissue, asterisk). The majority of papillary structures covered the tumor peripheral area (blue arrow) and in the center there are fibrous stroma and the secreted follicular structures that contain goiter (white arrow) (HE stain x 40, b). No tumor necrosis was seen and mostly rarity of mitosis. Immunohistochemistrical stain presents that tumor cells are positive with Thyroglobulin (immunohistochemistry stain x 400, d). Nuclear features of papillary thyroid carcinoma were not clear but poorly thyroid carcinoma and anaplastic thyroid carcinoma were no evidence. The images correlate with high-grade papillary thyroid carcinoma.

## Slide 4
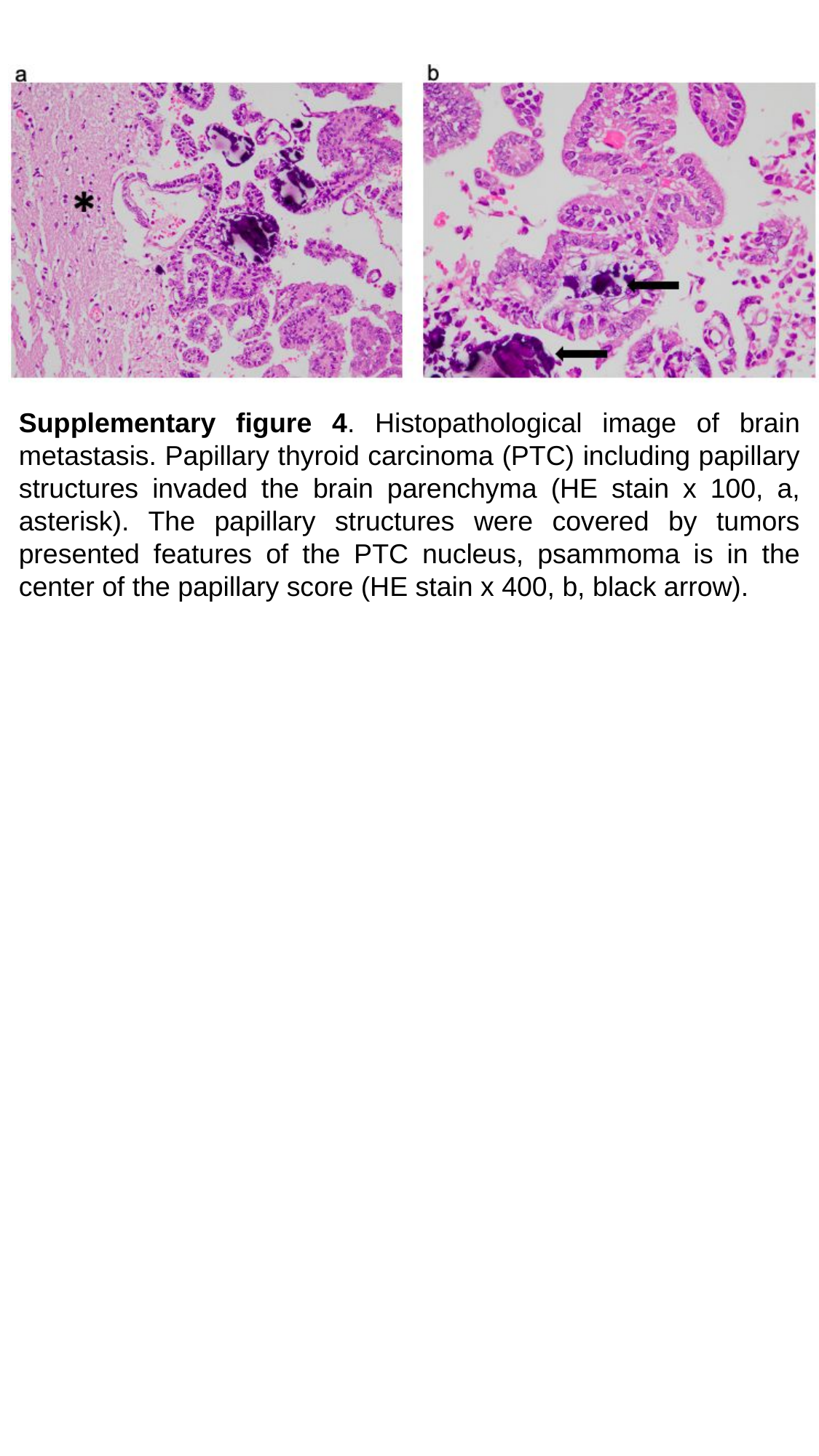

#
Supplementary figure 4. Histopathological image of brain metastasis. Papillary thyroid carcinoma (PTC) including papillary structures invaded the brain parenchyma (HE stain x 100, a, asterisk). The papillary structures were covered by tumors presented features of the PTC nucleus, psammoma is in the center of the papillary score (HE stain x 400, b, black arrow).
